# Polyamine Inhibition with DFMO: Shifting the Paradigm in Neuroblastoma Therapy

**DOI:** 10.3390/jcm14041068

**Published:** 2025-02-07

**Authors:** Joseph Schramm, Chloe Sholler, Leah Menachery, Laura Vazquez, Giselle Saulnier Sholler

**Affiliations:** Penn State College of Medicine, Penn State University, Hershey, PA 17033, USA; jschramm@pennstatehealth.psu.edu (J.S.);

**Keywords:** neuroblastoma, DFMO, LIN28, review

## Abstract

Neuroblastoma is a common childhood malignancy, and high-risk presentations, including an MYCN amplified status, continue to result in poor survival. Difluoromethylornithine (DFMO) is a new and well-tolerated treatment for high-risk neuroblastoma. This review article discusses preclinical and clinical data that resulted in the establishment of DFMO as a treatment for neuroblastoma. The review of preclinical data includes a summary of the contribution of polyamine synthetic pathways to high-risk neuroblastoma, the effect that MYCN has on polyamine synthetic pathways, and the proposed mechanism by which DFMO inhibits tumorigenesis. This understanding has led to the discussion of various preclinical combination therapies that may result in a synergistic therapeutic response for high-risk neuroblastoma. We review the clinical trials that show the successful treatment of high-risk neuroblastoma with DFMO, including comparative analysis and traditional neuroblastoma trials using propensity score matching. We review the regulatory path by which DFMO gained approval from the Federal Drug Administration for use as a maintenance therapy following the traditional high-risk neuroblastoma therapy. Finally, we discuss the role of DFMO in future clinical research for neuroblastoma and additional pediatric cancers.

## 1. Neuroblastoma Therapy

As our understanding of the biology of neuroblastoma (NB) has evolved, novel therapies targeting the biological underpinning have emerged. NB is cancer evolving from the neural crest cells of the developing parasympathetic nervous system. There are two peak incidences; the first, which is between birth and 18 months and is often termed infant NB, may spontaneously regress and is considered low risk with a very good prognosis. The second range is between 18 months and 6 years of age, and is often referred to as childhood NB; this is a more aggressive disease and, depending on the risk factors, is either intermediate or high risk. Given the good outcome for low- and intermediate-risk NB, the therapy for these has not changed significantly. Low-risk disease is treated with observation or surgery, while intermediate-risk disease is treated with 4–8 cycles of chemotherapy with surgery, each of these with >90% survival [1]. The children who present with high-risk neuroblastoma (HRNB) have a more challenging disease course. The treatment involves induction chemotherapy with surgery after cycle 4 or 5, followed by consolidation therapy including autologous stem cell transplant (single or tandem) and radiation therapy; this is followed by maintenance with anti-GD2 immunotherapy and retinoic acid [1,2,3,4]. The anti-GD2 immunotherapy was the first therapy to truly target the biology of NB. These tumors have been found to express the ganglioside GD2 on the cell surface. Anti-GD2 antibodies have been developed to prompt the immune system to recognize the tumor for immune clearance. This was the first biologically driven drug developed for NB and proven to increase survival [4,5].

With a deeper understanding of the biology of NB, newer agents have been explored. The genomic landscape of this disease has been studied, with MYCN amplification and ALK mutations being identified as potential targets [6,7]. Further research on the pathways involved in MYCN-driven tumors has highlighted the involvement of polyamine pathways [8]. In a review of the rate-limiting stop of this pathway, ornithine deoxycarboxylase (ODC) expression was shown to be a risk factor in NB, independent of MYCN amplification [9]. In addition, the drug difluoromethylornithine (DFMO) was identified as an inhibitor of this pathway. This review will focus on the polyamine pathway in cancer and the development of DFMO as a targeted medication for the treatment of HRNB. In preparation for this review article, we performed an overview of key literature by conducting PubMed searches using various combinations of the terms “DFMO”, “neuroblastoma”, “cancer”, “polyamine”, and “clinical trial” to provide a broad representation of published work on the topic.

## 2. Polyamines in Cancer

Polyamines, such as putrescine, spermidine, and spermine, are essential for cell growth and differentiation. They have been implicated in multiple cellular processes, including the stability of RNA transcripts, translation regulation, and the regulation of chromatin condensation. Their dysregulation has been linked to cancer development, as polyamines support the rapid proliferation of tumor cells [8]. As such, polyamine metabolism has become a promising target for therapy. Difluoromethylornithine (DFMO) was first synthesized and found, in 1978, to inhibit ornithine decarboxylase (ODC), which is responsible for converting ornithine to polyamines, the first step in the polyamine synthesis pathway [10,11]. DFMO has been utilized in vitro to reduce polyamine synthesis and inhibit tumor growth in various cancer models, including colon, breast, and prostate cancers [12,13,14]. ODC was discovered to be an important regulator of polyamine pathways in cancer and c-MYC was discovered to be an important regulator of ODC expression [15,16]. Polyamines have been implicated in the regulation of apoptosis in cancers [17]. In preclinical cancer models, it has been shown that DFMO reduces concentrations of putrescine, spermidine, and spermine, and decreases tumor proliferation.

## 3. Preclinical Research in NB

Difluoromethylornithine (DFMO), a selective inhibitor of ornithine decarboxylase (ODC), has shown promise in inhibiting NB tumorigenesis, particularly through its effects on the polyamine biosynthesis pathway. ODC overexpression is a hallmark of NB, contributing to increased cell proliferation and tumor progression, and correlates with poor survival [9,18]. MYCN amplification is present in 30% of NB cases and is associated with aggressive tumor growth. MYCN upregulates polyamine synthesis through the multiple mechanisms discussed below. DFMO was studied early in neuroblastoma research, prior to understanding the influence that polyamines have on cancer regulation [19]. MYCN was later described as an important regulator of ODC1 in neuroblastoma [9].

Pre-clinical models using human neuroblastoma samples and cell lines also show that MYCN amplification results in an increased expression of polyamine synthetic enzymes (ornithine decarboxylase antizyme inhibitor (OAZIN), spermidine synthase (SRM), spermine synthase (SMS), and adenosylmethionine decarboxylase 1 (AMD1)), and a decreased expression of catabolic enzymes (ornithine decarboxylase antizymes (OAZs), spermidine/spermine N1-acetyltransferase (SAT), and spermine oxidase SMOX) [9,20,21]. Human neuroblastoma cell lines also show decreased growth and maturation when treated with DFMO, although some cell lines without MYCN amplification show a reduced response compared to those with MYCN amplification [9,16]. DFMO treatment results in a decrease in polyamine concentrations. In preclinical neuroblastoma models, DFMO results in a remarkable decrease in the concentration of putrescine and a variable decrease in spermidine, but the spermine levels are usually unaffected.

Spermidine is responsible for the hypusination of elongation initiation factor 5a (eIF5a) [22]. DFMO results in the decreased hypusination of eIF5a in preclinical neuroblastoma models [23,24,25], and hypusine dysregulation is associated with poor prognosis [26]. eIF5A has been shown to be an important regulator of translation in oncologic processes. The DFMO-mediated regulation of eIF5a also results in an alteration of the LIN28/LET7 pathway. LIN28 is an RNA-binding protein that is associated with cell survival and stemness in cancer [27,28,29]. LET7 is a miRNA that inhibits LIN28 activity. MYCN is a known inhibitor of LET7, and MYCN-amplified neuroblastoma has an increased expression of LIN28 [30,31]. DFMO treatment in preclinical neuroblastoma models results in decreased LIN28 expression [32]. LIN28-mediated glycolytic metabolism is also decreased with DFMO therapy. Additionally, neuroblastoma cell lines with decreased LIN28 expression following DFMO treatment have an impaired capacity to form neurospheres and decrease tumor initiation [33].

When DFMO dosing was used at physiologically feasible concentrations to treat five different established human NB cell lines (three of five with MYCN amplification), the cell viability was decreased, and apoptosis was significant at 48 and 72 h when compared to the vehicle controls. DFMO induced cell-cycle arrest, particularly at the G1/S transition, and a 6-fold increase in beta-galactosidase (SA-BGal) activity, a marker of cellular senescence. This arrest, coupled with a reduction in cyclin D1 and phospho-Rb levels, suggests that DFMO interferes with the proliferative capacity of NB cells [32]. These findings support that DFMO has cytostatic, as opposed to cytotoxic, effects in cancer cells.

Neuroblastoma forms neurospheres in cell culture, which are associated with increased cancer stems cells and tumor initiation [33]. In neurosphere assays on the same five human neuroblastoma cell lines that were treated with DMFO at physiologically relevant concentrations, the formation of neurospheres was inhibited in all cell lines. However, MYCN amplification cell lines required higher doses of DFMO to have comparable results. The ability of DFMO to inhibit neurospheres and tumor initiation increases with the DFMO concentration and treatment duration, making its clinical use appealing.

In vivo, the pretreatment of neuroblastoma cells with DFMO prior to injection inhibited neurosphere formation in mice. In addition, DFMO treatment inhibited tumor formation in limiting dilution assay (LDA) xenograft models [33]. The mice injected with minimal disease using MYCN-amplified NB cells (BE2C and SMS-KCNR) treated with DFMO in drinking water exhibited delayed tumor formation, a reduced tumor volume, and improved event-free survival [33]. Moreover, the tumor cells from DFMO-treated mice showed a reduced expression of MYCN and LIN28B, further supporting the hypothesis that DFMO inhibits tumorigenesis by targeting these critical oncoproteins. This evidence suggests that DFMO disrupts tumor progression, making it a potential therapeutic strategy for HRNB.

Mice models with abdominal neuroblastoma tumors resulting from neural-crest-specific MYCN transgenic expression (TH-MYCN mice) have been used to study neuroblastoma. TH-MYCN mice with the heterozygous knockout of ODC1 did not have improved survival. However, the DFMO inhibition of ODC improved survival [9,34]. TH-MYCN had increased survival and synergy when DFMO was administered in combination with cisplatin, vincristine, and cyclophosphamide. The cyclophosphamide resulted in the long-term curing of 20% of mice, whereas the DFMO/cyclophosphamide combination resulted in the long-term curing of 80% of mice [9].

Dietary arginine and proline are the primary sources of the circulating polyamines that are imported into TH-MYCN neuroblastoma tumors and can result in DFMO resistance [35]. The combination of DFMO with the dietary restriction of arginine showed a decrease in eIF5a-mediated translational regulation, which resulted in ribosomal stalling for genes that control the cell cycle and proliferation and improved the translational expression of genes that regulate neuronal differentiation. Under the influence of DFMO, neuroblastoma increases intracellular polyamine importation [34]. Various strategies have been used to divert polyamines from accumulating via alternative pathways and intracellular import.

TH-MYCN mice were treated with both DFMO and AMD1 inhibitors to reduce polyamine synthesis. The upregulation of other polyamine synthetic enzymes may also result in resistance to DFMO. The AMD1 inhibitor SAM486 resulted in an additive effect when combined with DFMO. Although SAM486 resulted in the accumulation of putrescine, tumor cells had a higher concentration of spermidine, likely resulting from the import of extracellular polyamines [36].

When DFMO was combined with the COX inhibitor celecoxib, a synergistic effect was observed. COX inhibition results in improved SAT1 expression, which acetylates polyamines and results in the exportation of polyamines out of the cell. This opposes the cellular importation of circulating polyamines, which is a primary mechanism of resistance to DFMO. The combination of DFMO and celecoxib resulted in decreased spermidine when compared to SAM486. The survival of TH-MYCN mice and mice with human neuroblastoma xenograft improved with the addition of chemotherapy, including cyclophosphamide [36].

**Figure 1:**
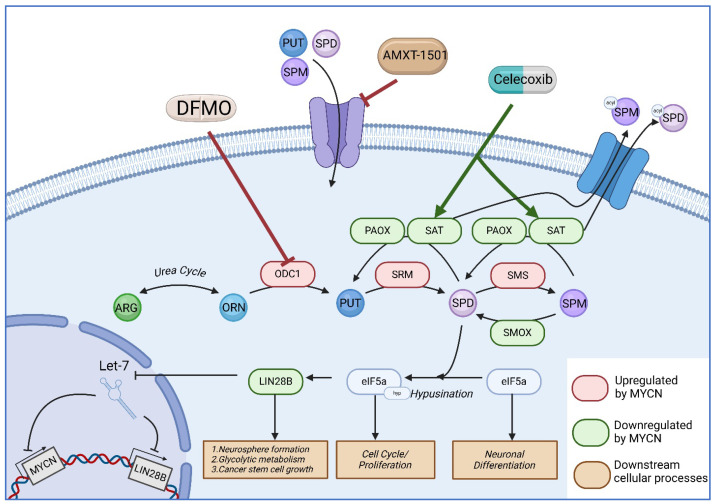
Graphical summary of polyamine metabolism in neuroblastoma abbreviations: acyl—acyl group, ARG—arginine, DFMO—difluoromethylornithine, eIF5a—elongation initiation factor 5a, hyp—hypusine group, Let-7—lethal-7 micro RNA, LIN28B—Lin-28 homolog B, MYCN—N-myc, ODC1—ornithine decarboxylase, ORN—ornithine, PAOX—polyamine oxidase, PUT—putrescine, SAT—spermidine/spermine acetyltransferase, SMOX—spermine oxidase, SMS—spermine synthase, SPD—spermidine, SPM—spermine, SRM—spermidine synthase.

When DFMO was combined with a polyamine transport inhibitor, AMXT 1501, to further address resistance to ODC1 inhibition, this combination resulted in the decreased growth of neuroblastoma cell lines in vitro and in vivo for TH-MYCN transgenic mice [34,37]. The combination of AMXT 1501 and DFMO resulted in decreased putrescine and spermidine. Spermine remained unchanged. DFMO/AMXT 1501 showed synergy in the treatment of neuroblastoma in TH-MYCN transgenic mice when combined with irinotecan/temozolomide and cyclophosphamide/topotecan [34]. Please refer to Figure 1 for a graphical representation of the preclinical research described above.

## 4. Clinical Trials

### 4.1. Clinical Trials—Phase I

The primary goal of the phase I clinical trial was to study the safety of DFMO alone and in combination with etoposide, a cytotoxic chemotherapeutic drug, in pediatric patients with refractory or recurrent NB. Etoposide was the chemotherapy drug as it was shown to be synergistic with DFMO in NB cell models [38]. The secondary goal was to investigate the activity, pharmacokinetics, and genetic and metabolic factors associated with ODC. DFMO was administered alone for three weeks, followed by additional three-week cycles of DFMO with daily oral etoposide for 14 days of each cycle. Four doses of DFMO were evaluated and its safety was confirmed, with no dose-limiting toxicities (DLTs) identified in subjects taking between 500 and 1500 mg/m^2^ of DFMO orally twice a day [39]. Six of the eighteen evaluable subjects were without progression during the trial period, and three subjects were progression-free > 5 years after completing treatment.

Subjects with the minor T-allele at rs2302616 of the ODC gene had higher baseline levels (*p* = 0.02) of, and larger decreases in, total urinary polyamines during the first cycle of DFMO therapy (*p* = 0.003) and had a median progression-free survival (PFS) over three times longer than subjects with the major G allele at this locus; however, this result was not statistically significant (*p* = 0.07).

The phase I trial also included work that evaluated the use of high-dose DFMO (maximum tolerated dose was 6750 mg/m^2^/day) with celecoxib, cyclophosphamide and topotecan in refractory neuroblastoma [40]. In total, 63% of patients had a response to treatment, 13% remained progression free 4 years after the completion of therapy without additional treatment, and 8% had dose-limiting toxicity.

### 4.2. Clinical Trials—Phase II

A single-arm Phase II open-label, single-agent, multicenter clinical trial for subjects with HRNB who had completed standard therapy or therapy for refractory/relapsed disease was performed by the Beat Childhood Cancer research consortium, NMTRC003/003B, from 2012 to 2016 [41]. The primary endpoint was EFS from the first dose of DFMO, and the secondary objectives included OS and safety. Patients were enrolled in two strata, the first being patients achieving the completion of immunotherapy for HRNB, and the second being patients with relapsed or refractory HRNB who had achieved no evidence of disease (NED). The subjects in Stratum 1 showed a two-year EFS of 84% ± 4% and OS of 97% ± 2%, while the subjects in Stratum 2 exhibited a two-year EFS of 51% ± 8% and OS of 84% ± 6%.

DFMO was well tolerated as maintenance therapy, with very few Grade 3 and 4 toxicities. The only Grade 4 toxicity reported was hypoglycemia (1%) [41]. The most frequent Grade 3 toxicities included neutropenia (4%), elevated transaminases (4%), and hearing loss (4%). The Grade 2 toxicities were similar to the Grade 3 toxicities, and any singular Grade 2 toxicity was seen in 5% or less of participants. Since clinical trials have been primarily used as maintenance therapy following traditional neuroblastoma therapy or as treatment in post-relapse/refractory disease therapy, the effect of DFMO in relation to surgery or radiation therapy has not been studied to date.

To further analyze these results, a subset analysis from this trial compared the event-free survival (EFS) and overall survival (OS) outcomes of 81 subjects receiving DFMO after standard induction and consolidation therapy to those of a historical cohort of 76 subjects treated at hospitals affiliated with the Beat Childhood Cancer Research Consortium (BCC), using the same upfront therapy but without DFMO [42]. The results showed that the 2-year and 5-year EFS was significantly higher for the DFMO group (86.4% and 85.2%, respectively) compared to the historical cohort (78.3% and 65.6%). Similarly, the 2-year and 5-year OS was better in the DFMO group (98.8% and 95.1%) than the control group (94.4% and 81.6%). This suggests that maintenance therapy with DFMO may provide a clinically significant benefit for HRNB subjects, particularly in terms of long-term survival.

The mechanisms behind DFMO’s effectiveness through the inhibition of ornithine decarboxylase (ODC) is by reducing tumor progression and carcinogenesis. Additionally, DFMO’s effect on MYCN, often amplified in HRNB and associated with poor prognosis, may contribute to its therapeutic efficacy. In this analysis, subjects with MYCN amplification who received DFMO showed a statistically significant improvement in OS at 5 years, from 78.1% to 97.3%, highlighting DFMO’s potential to address higher-risk subgroups with HRNB.

The study design included patients enrolled at 15 BCC hospitals between June 2012 and February 2016, and enrolled patients up to 120 days before the end of therapy. To mitigate bias, only subjects who were treated during this time period and those remaining event-free 120 days after completing standard therapy were included in a multiple sub-analysis, ensuring comparability between the DFMO-treated group and the historical cohort. While minor differences in age and induction response were noted between the two groups, the statistical analyses showed the clear advantage of DFMO treatment in both EFS and OS [42]. A further exploration of the role of DFMO in preventing early relapse, particularly when starting during immunotherapy, and its potential impact on other molecular targets, such as c-MYC, may further enhance its therapeutic potential for HRNB. Please refer to Table 1 to determine EFS and OS from the analysis described above.

### 4.3. FDA Approval

The significant effect seen in the initial Phase II study led to a unique approval path with the FDA. In 2020, Breakthrough Therapy Designation was awarded, which led to frequent communication and collaboration between the agency and research team. The regulatory framework regarding rare diseases shifted, allowing for an analysis of the data and the identification of an appropriate fit for external control. This was identified, as most patients enrolling in the NMTRC003/003B study had previously been enrolled in and completed the ANBL0032 clinical trial within 120 days. The raw data from this database were shared by the Children’s Oncology Group. The subjects who had enrolled in NMTRC003/003B were removed, as well as the remaining subjects who would not have been eligible for enrollment in the NMTRC003/003B trial. After this was completed, 850 subjects remained for comparison. With these subjects, a propensity score matched (PSM) analysis was conducted. PSM analysis is a statistical method used to match subjects from two datasets based on a propensity score and reduce biases when comparing patient data from different sources [43,44,45]. It matches individual subjects based on propensity scores to optimize the similarity of compared groups and therefore balances covariates between treated and control subjects. If has been shown to reduce confounding differences to better isolate the treatment effect for evaluated outcomes. The variables utilized in the PSM analysis included an identical match for MYCN status, and a PSM score match based on the following: age at high-risk diagnosis, race, sex, stage, pre-transplant response, transplant type, time from transplant to the start of immunotherapy, time from the start to the end of immunotherapy, and overall response at end of immunotherapy. The data were submitted to the statistical groups for analysis only after the variables were defined and agreed upon by the FDA, US World Med, and the Beat Childhood Cancer Research Consortium (BCC) leadership. The data showed statistical significance, showing an improvement in both EFS and OS (See Table 1) [46].

While standard drug approval in oncology requires a randomized control trial, this data enabled us to evaluate whether it was ethical to perform such a study at this time in a rare pediatric cancer with historically poor outcomes, showing an increase in OS of 12% at 4 years. We considered the length of time it would take to complete a randomized trial in comparison to the number of lives lost, given the lower OS in the standard arm. Given that this would be the first oncology drug approved with a single-arm clinical trial, external validation and review were requested, with an Oncology Drug Advisory Committee (ODAC) meeting held on 4 October 2023 [47]. The regulatory framework within the FDA at this time allowed for the evaluation of a drug in a single-arm study if it met the criteria of having an appropriate external control, an adequate and well-controlled study, substantial confirmatory evidence, and an overall risk–benefit analysis. Each of these were reviewed at the ODAC meeting. The final voting question, “Has the applicant provided sufficient evidence to conclude that DFMO improves EFS in patients with HRNB?”, resulted in an affirmative vote by 70% of the panel. This resulted in the FDA approval of DFMO on 13 December 2023 due to its ability to reduce the risk of relapse in adult and pediatric patients with HRNB who have demonstrated at least a partial response to prior multiagent, multimodality therapy, including anti-GD2 immunotherapy.

## 5. Discussion and Future Directions

DFMO has been shown to be an efficacious, well-tolerated, and safe addition to therapy for HRNB. The FDA approval of DFMO for patients with HRNB has resulted in its wide adoption in the standard of care, changing the current paradigm for NB care to now include an extended 2-year post-immunotherapy maintenance to decrease the previous high rate of relapse. Further study will help to answer additional questions regarding the optimal dosing, additional uses in therapy and ideal combination therapies. Additional ongoing studies include the NMTRC014, which is a BCC-sponsored trail that is currently evaluating the differential dosing of DFMO for use in maintenance settings. NMTRC012, another BCC-sponsored trial, is evaluating the efficacy of DFMO when used earlier in treatment during immunotherapy, using a randomized control schema. Both studies are aiming to further reduce the frequency of relapses in first-line therapy for HRNB.

There is promising preclinical research showing that DFMO in combination with other interventions can overcome resistance to the ODC1 inhibition of polyamine synthesis. These combination therapies investigate the impact of diet, polyamine transportation, and intracellular polyamine trafficking. Some of the preclinical work has resulted in the development of clinical trials [40]. AMXT1510 in combination with DFMO has yet to be studied in the clinical setting in pediatrics, but a trial for this combination is being developed.

Lastly, there are many other childhood tumors that are expected to be highly impacted by the MYCN, ODC1, and LIN28/Let7 pathways, such as diffuse intrinsic pontine glioma (DIPG), atypical teratoid/rhabdoid tumor (ATRT), embryonal tumor with multilayer rosettes (ETMR), medulloblastoma, Ewing Sarcoma, osteosarcoma, and rhabdomyosarcoma. Expanded access to DFMO, in order to evaluate the efficacy of DFMO in relapsed and refractory rare pediatric tumors, is currently available through the clinical trial NMTRC006B. We expect further research and additional trials will open soon to further support the mission of curing childhood cancers.

## Figures and Tables

**Table 1:** Sequential analyses of NMTRC 003/003B trial reported between 2018–2023.

Analysis 2018	N	Comparison Data	N	EFS—2 year	EFS—4 year	OS—2 year	OS—4 year
Stratum I	100			84 ± 4%	83 ± 4%	97 ± 2%	96 ± 2%
MYCN amp. (+) vs. MYNC amp. (−)	39			85 ± 5% vs. 86 ± 5%	85 ± 5% vs. 83 ± 5%	98 ± 2% vs. 96 ± 3%	98 ± 2% vs. 94 ± 4%
Stratum II	39			51 ± 8%	44 ± 9%	84 ± 6%	62 ± 11%
**Analysis 2020**				**EFS—2 year**	**EFS—5 year**	**OS—2 year**	**OS—5 year**
Stratum I subset	81	Historical cohort: HRNB	76	86.4% vs. 78.3%	85.2% vs. 65.6%	98.8% vs. 94.4%	95.1% vs. 81.6%
Sub−analysis: MYCN amp. (−)	41	Historical cohort: HRNB MYCN amp. (−)	39	82.9% vs. 81.3%	80.5% vs. 67.3%	97.6% vs. 97.3%	92.7% vs. 88.7%
Sub-analysis: MYCN amp. (+)	27	Historical cohort: HRNB MYCN amp. (+)	30	91.9% vs. 86.4%	91.9% vs. 75.6%	100% vs. 92.9%	97.3% vs. 78.1%
**Analysis 2023**		**Comparison**			**EFS—4 year**		**OS—4 year**
Stratum I	92	ANBL0032 Trial	852		84 ± 4% vs. 72 ± 2%		96 ± 2% vs. 84 ± 1%
1:3 PSM Stratum I	90	1:3 PSM ANBL0032	270		84 ± 4% vs. 73 ± 3%		96 ± 2% vs. 84 ± 2%
1:3 PSM Stratum I: CR at EOI	88	1:3 PSM ANBL0032: CR at EOI	264		84 ± 4% vs. 74 ± 3%		95 ± 2% vs. 86 ± 2%
1:2 PSM Stratum I: Contemporary	91	1:2 PSM ANBL0032: Contemporary	182		84 ± 4% vs. 72 ± 3%		96 ± 2% vs. 84 ± 3%

Abbreviations. ANBL0032—Children’s Oncology Group Neuroblastoma Trial ANBL0032, CR—Complete Remission, EOI—End of Induction, HRNB—High Risk Neuroblastoma, PSM—Propensity Score Matching.
